# The Role of Outpatient Intravenous Diuretic Therapy in a Transitional Care Program for Patients With Heart Failure: A Case Series

**DOI:** 10.4021/jocmr1106w

**Published:** 2012-11-11

**Authors:** Mohamad Lazkani, Ken S. Ota

**Affiliations:** a Department of Transitional Care Medicine, Banner Good Samaritan Medical Center; Phoenix, AZ, USA

**Keywords:** Outpatient parenteral diuretic therapy, Intravenous, Transitionalist, Transitional care, Heart failure, Fluid overload, Outpatient diuresis, Furosemide, Metolazone

## Abstract

We present a case series of seven patients with an established diagnosis of heart failure enrolled in a transitional care program that were treated with intravenous diuretic therapy in the outpatient setting. The patients presented in this cases series were treated due to the development of decompensated heart failure within 30 days of their discharge from our hospital. All seven patients stated that they would have originally presented to the emergency department for their symptoms, but consented to alternative treatment by a transitional care physician, or transitionalist. These patients with decompensated heart failure (four male and three female) with a median age of 55 years (24 - 84 years) were evaluated and treated from November 2011 to March 2012. Of the seven patients, four presented with decompensated systolic heart failure (three with diastolic). All seven patients were treated with an intravenous diuretic for hypervolemia in our outpatient infusion room. All of the patients experienced relief of their dyspnea the day of diuretic administration or the following day. No adverse effects or emergency department transfers occurred as a result of outpatient intravenous diuretic therapy. Through the use of outpatient intravenous diuretic therapy, we have avoided the need for emergency department visits and potential hospitalization in select patients with decompensated heart failure. Based on our preliminary findings, the clinical efficacy of this approach to the treatment of heart failure decompensation is not only due to the pharmacologic effectiveness of intravenous diuretics, but also due to the bidirectional open line of communication that exists between transitionalist and patients in the program. The direct telephone access that patients have to the transitionalist allows for close post-hospitalization monitoring and facilitates timely evaluation and treatment when acute issues arise. The added benefit of our particular transitional care program is that we have an alternate venue in the hospital where our transitional care patients can be treated for heart failure decompensation (our outpatient infusion room), thus, enabling us to avoid emergency department transfers and possible hospital admissions. Further investigation of this therapy in a non-emergency department setting is warranted as our experience with this treatment modality is limited to the case series presented.

## Introduction

Heart failure (HF) has a prevalence of over 5.8 million in the USA and over 23 million worldwide. This is increasing due to the aging population, and improved management of heart disease, although the age-adjusted incidence has plateaued [1]. HF continues to be a tremendous burden to the healthcare system, despite advances in management. Patients with acute HF exacerbations present to the emergency department (ED) and are often admitted. Exact figures are unclear, but one Canadian study suggests that this number is remarkably high, accounting for 10,415 acute HF patients ≥ 65 years of age who presented to the ED between 1998 and 2001. They found that 65% of these patients were admitted to the hospital [2].In the US, HF accounted for 727,000 ED visits, and 1 million hospital discharges in 2003 [3, 4]. It has been estimated that hospitalizations for HF has tripled over the span of 25 years from 1.27 million to 3.86 million from 1979 to 2004 [5]. Unfortunately, the management of chronic HF may become quite complex, requiring constant surveillance and medical attention. One of the challenges of controlling HF symptoms is maintaining proper fluid-balance during a patient’s post-hospitalization period. A recent study of Medicare claims data from 2003 to 2004 found that HF was the leading cause of hospital 30-day readmission, surpassing all other medical and surgical conditions [6].

In response to the burden of hospital readmissions and emergency department (ED) visits, there have been several publications of transitional care strategies that address the post-hospitalization needs of HF populations and their ability to mitigate readmission risk [7-11]. At our institution, we have also addressed this issue by developing a physician-led transitional care medicine program. This medical consulting service aims to: 1) improve the post-hospitalization care transition process for patients admitted for HF decompensation; 2) increase access to care by providing personalized, physician-delivered medical services during the post-hospitalization period via direct patient-physician telephone access, home visits, and skilled nursing facility visits; 3) improve the fluidity of information transfer between the inpatient sector and the patients’ respective community physicians or help patients establish with a primary care physician (PCP) if they do not have one; and 4) reduce the need for all-cause ED visits and/or 30-day rehospitalizations. We use the term “transitionalist” to address the physician coordinating and leading this endeavor [9].

Our goal in this analysis is to present our experience in treating patients with evidence of HF decompensation through our transitional care program with the use of intravenous diuretic therapy in a non-ED setting: our outpatient infusion room (OIR).

## Cases

Inpatient physicians and case managers refer patients admitted for HF decompensation to the transitional care program. The transitionalist, in collaboration with the patient and the respective inpatient case manager, determines transitional care needs and develops the post-hospitalization care plan at the bedside, while patients are still in the hospital. Each patient enrolled into the program is seen by the transitionalist within 24 to 72 hours for follow-up in his or her community (residence or post-acute care facility). Patient education regarding HF self-care and special instructions are provided at the time of discharge to patients enrolled in our program and are reiterated at the follow-up appointment. During the post-hospitalization period, if a patient has complaints of weight gain (three or more pounds), increased dyspnea, and/or increased edema, they are instructed to do a trial of up-titration of oral diuretic therapy for 24 hours (based on the direction of the transitionalist). If there is no improvement, they are then physically evaluated by the transitionalist. If a patient has subjective complaints of shortness of breath, they are seen by the transitionalist the same day. Patients are instructed to present to the OIR of the hospital where the transitionalist can conduct evaluation. The patients are discharged home once they feel that their dyspnea has resolved and if deemed safe for such a disposition. Patients that do not improve clinically can opt for reinitiating IV diuretic therapy the following day as an outpatient. It is understood, a priori, that if symptoms worsen, the patient will be transferred to the ED or directly admitted to the hospital. Moreover, if the transitionalist deems it necessary, the patient will be instructed to proceed to the ED from home.

The transitionalist’s decisions on how to treat our patients in the OIR are based on clinical judgement. If it is determined that there is a compelling reason to abandon this type of therapy, there is liberty to send the patient to the ED (i.e. suspicion for acute life threatening illness). However, none of the cases in this series were deemed necessary for ED transfer by the transitionalist. Furthermore, anyone with a history of chronic kidney disease who is a candidate for intravenous therapy has a basic metabolic panel drawn prior to therapy to ensure that they are not in overt acute renal failure (ARF). Patients with ARF are excluded from outpatient intravenous therapy and opted for inpatient hospital care. None of the patients treated had ARF or were hemodynamically unstable.

From November 2011 to March 2012, four patients with decompensated systolic HF and three with decompensated diastolic HF (four male and three female) with a median age of 55 years (24 - 84 years) were treated as outpatients with intravenous diuretic therapy as they presented with evidence of hypervolemia. All treatments included loop diuretics (intravenous furosemide or bumetanide) and were used with five mg of oral metolazone concomitantly. The IV dose of diuretic that was administered was based on the transitionalist’s clinical judgement, as would be done in any standard clinical encounter. All patients received additional oral potassium chloride supplementation. The dose of potassium was at the discretion of the transitionalist based on the dose of diuretic given.

Laboratory and imaging studies were ordered only if the transitionalist deemed such orders clinically necessary. As the diagnosis of acute HF is a clinical diagnosis and the patients in the transitional care program were well-known to the transitionalist, many of the studies that may be commonly ordered in the ED setting were avoided. Table 1 presents patient- and treatment-specific characteristics.

**Table 1:** Transitional Care Program Patients Receiving IV Diuretic Therapy in the Outpatient Infusion Room

Patient	Sex	HF Type	NYHA Class	Diuretic	Duration of Treatment (Days)	Notes
1	Male	Systolic	3	Day 1: Furosemide	2	Improved dyspnea on the 2nd day of treatment
				Day 2: Bumetanide		
2	Male	Systolic	3	Furosemide	1	Dyspnea resolved after 8 hours of treatment
3	Male	Systolic	3	Furosemide	1	Dyspnea resolved after 12 hours of treatment
4	Female	Diastolic	3	Furosemide	1	Dyspnea resolved after 12 hours of treatment
5	Female	Diastolic	3	Furosemide	1	Dyspnea resolved after 12 hours of treatment
6	Male	Systolic	3	Furosemide	1	Dyspnea resolved after 12 hours of treatment
7	Female	Diastolic	3	Bumetanide	1	Dyspnea resolved after 8 hours of treatment

## Results

A total of seven patients were treated with outpatient intravenous diuretic therapy. All patients treated in the outpatient infusion room remained hemodynamically stable throughout the therapeutic course and maintained normal blood pressures while being continuously monitored. Six of the seven patients were discharged home after one day (maximum 12 hours) of diuretic therapy as they were clinically improved. One patient required a second day of diuretic therapy as he did not have significant clinical improvement of his dyspnea after one day of treatment with IV furosemide. He was treated the subsequent day with IV bumetanide and responded well. He was discharged home after the second round of diuretic therapy. All patients were contacted by the treating physician via telephone at the end of the business-day and again the next day for follow-up. All seven patients were instructed to follow-up with their cardiologists within one week after diuretic therapy to determine if an adjustment in their home diuretic regimen was necessary. None of the seven patients had a subsequent episode of HF decompensation within the 30-day post-hospitalization period. The precipitating factor for HF exacerbation was either due to high consumption of sodium-containing food or poor medical regimen adherence.

## Discussion

Transitional care has been defined as a set of actions designed to ensure the coordination and continuity of care received by patients as they transfer between different locations or levels of care [11]. Our goal, through the transitional care program at our institution, is to optimize post-hospital care of patients with HF who are considered high-risk for rehospitalization within 30 days. At our institution, we do not use rehospitalization risk assessment tools as their performance in accurate risk stratification is sub-optimal [12]. We consider every patient admitted for acute HF as “high-risk” and, therefore, offer transitional care services to them. We support, however, that the timeliness of post-discharge follow-up, proper coordination of care with the respective community physicians, and detailed medication reconciliation may decrease rehospitalization risk in our patients [12].

Various transitional care strategies that address the post-hospitalization needs of the HF population and their ability to mitigate readmission risk have been described [7-11]. One recently published prospective study highlights preliminary results that suggest the transitional care program performed at their institution reduced the 30-day readmission rate by almost 50% for patients with HF [8]. Another program designed and implemented through a joint collaboration between a certified home healthcare agency and regional hospital has demonstrated providing transitional care services to high-risk HF patients can be an effective deterrent against patterns of rehospitalization [10]. Finally, a randomized controlled trial that included elderly patients with HF, demonstrated that a comprehensive transitional care program increased length of time between hospital discharge and readmission or death, reduced total number of rehospitalizations, and decreased healthcare costs [7].

Interestingly, the comprehensive strategy used in our transitional care program for patients with HF [9], appears to be contributing to decreased ED visits and rehospitalizations at our hospital as well (unpublished data at the time of this publication). Through the use of outpatient IV diuretic therapy in our OIR, we have been able to eliminate the need for ED visits (and potential hospitalizations) in select patients with decompensated HF that are actively enrolled in our transitional care program. Although we cannot determine with absolute certainty that our symptomatic patients would have been admitted to the hospital, all patients expressed that they would have presented to the ED had such a therapeutic option not been available.

We believe our strategy has been effective with the use of the OIR as a non-ED venue for HF treatment in conjunction with bidirectional telephonic communication between patients and the transitionalist. The availability of the OIR to evaluate and manage patients with HF decompensation specifically allows us to capture and treat patients that become symptomatic despite all other efforts within the transitional care services.

Outpatient IV diuretic therapy for HF has been described and is suggested that it can decrease emergency department visits and reduce HF readmissions [5, 13]. Interestingly, Hebert K et al [5] did not obtain blood chemistry panels prior to intravenous therapy and deemed this practice as safe in their population. In general, we support this notion, however, we decided to obtain basic metabolic panels prior to administering therapy in patients with chronic kidney disease, as one of the transitionalist’s exclusion criterion to outpatient IV therapy is ARF. In these cases, we would opt for inpatient hospital care, as this may complicate acute HF care. We do not believe that every patient with HF decompensation necessarily requires laboratory studies and imaging.

Although treatment is tailored to individual patient needs based on their history and physical exam, the preference in our program is to administer primarily furosemide in addition to a one-time dose of metolazone. Oral potassium supplementation occurs during all treatment sessions unless there is a clear contraindication. Review of the literature demonstrates that the combination of low-dose metalozone and loop diuretics are effective in most refractory HF patients in reducing edema with low incidence of electrolyte disturbances [14]. One may argue that the diuretic effect may be attributed to either the metolazone or the loop diuretic alone, however, we hypothesize it is the mechanistic synergy of the two drug classes that makes this regimen superior to either one alone.

Our current practice is to perform outpatient IV diuretic therapy only in hemodynamically stable patients that are enrolled in the transitional care program. This mode of therapy may have cost-avoidance implications to hospitals that care for HF populations. A preliminary review of our financial data reveal that the cost for implementing diuretic therapy in the OIR for one day is approximately $200, while the cost of implementing similar treatment in the inpatient setting is approximately $2,000 for one day. Assuming that all seven of these patients would have been admitted to the hospital for acute HF (under the same conditions with resolution of symptoms as we observed with our case series), eight patient-days of treatment would amount to $16,000 of cost, whereas, eight patient-days of treatment in the OIR would be only be 10% of the inpatient cost. By targeting appropriate patients with HF decompensation that are candidates for outpatient therapy, it appears that there may be significant cost-avoidance if they are managed as we have described in this case series. We aim to re-examine the clinical and economic impact of this therapy in a future communication as we accumulate more data.

### Conclusion

Our transitional care program aims to optimize the post-hospitalization care of patients with HF. The transitionalist is closely involved in the care of patients starting at the peri-discharge period, in order to provide frictionless continuity during the inpatient-to-outpatient transition. Our findings regarding the clinical efficacy of outpatient IV diuretic therapy in patients with HF decompensation are consistent with the limited data available in the peer-reviewed literature. We have demonstrated the safety and efficacy of this mode of therapy without any adverse events in a small sample of patients. Through our program, patients enjoyed HF-specific acute therapy without the need for ED visits or potential hospital admissions. Recognizing the tremendous burden HF places on the healthcare system, outpatient IV diuretic therapy in a non-ED setting may be an important component of any transitional care program’s armamentarium. Although it is not possible to draw any firm conclusions from our case series, we believe that our data suggests the potential utility of outpatient IV diuretic therapy in treating hemodynamically stable patients with HF decompensation and hypervolemia. Further studies are needed to clearly assess how this new level of care can impact our healthcare system both clinically and economically and how it might improve patients’ quality of life by avoiding unnecessary hospitalizations.
